# Supplementary material for: Melbournevirus encodes a shorter H2B-H2A doublet histone variant that forms structurally distinct nucleosome structures
Source: Nat Commun. 2025 Jul 26;16:6903. doi: 10.1038/s41467-025-62031-2 (PMC12297534; doi:10.1038/s41467-025-62031-2)
Supplement: Supplementary file 1 — Supplementary Information [file 41467_2025_62031_MOESM1_ESM.pdf]

Supplementary Information:

Tables:

Supplementary Table 1: Identity percentage of mel_149 compared to other melbournevirus (MV) histones, and to *Xenopus laevis* (*X. laevis)* and *Acanthamoeba castellanii* (*A. cast)* histones. Note the lack of any identity between the H2B portion of MV-varH2B-H2A and other H2B sequences.

	mel_149 (A0A097I1R9)					
	Total protein sequence		H2B region		H2A region	
Protein (Uniprot code)	Blast search (alignment/ total length)	Full sequence alignment	Blast search (alignment/ total length)	Full sequence alignment	Blast search (alignment/ total length)	Full sequence alignment
MV H2B-H2A (A0A097I2B5)	31% (55/176)	20%	non-significant	20%	39% (48/82)	20%
MV H4-H3 (A0A097I2D0)	23% (17/74)	12%	-	-	-	-
A. cast H2B (L8H4F8)	-	-	non-significant	9%	-	-
A. cast H2A (L8GN90)	-	-	-	-	33% (42/86)	18%
X. laevis H2B (P02281)	-	-	20% (19/79)	10%	-	-
X. laevis H2A (P06897)	-	-	-	-	35% (46/81)	22%

Supplementary Table 2: MV-varNLP cryo-EM data statistics

**Data collection and processing**	
Magnification	130000
Voltage (kV)	300
Electron exposure ($e^-/Å^2$)	50
Defocus range (µm)	1-20
Pixel size (Å)	0.97
Symmetry imposed	C1
Initial particle images (#)	1,010,995
Final particle images (#)	36,051
Map resolution (Å)	4.41
FSC Threshold	0.143
Map resolution range	3.991-58.21
**Refinement**	
Initial model used	AlphaFold 2 Prediction
Map sharpening B factor ($Å^2$)	147.5
Model Composition	
Non-hydrogen atoms	8897
Protein residues	655
Nucleotide	182
Ligand	0
B factors ($Å^2$)	
Map	147.5
Protein (min / max / mean)	107.05 / 297.53 / 177.88
Nucleotide (min / max / mean)	177.44 / 470.71 / 242.31
Ligand	N/A
R.M.S deviations	
Bond lengths (Å)	0.004 (0)
Bond angles (°)	0.716 (1)
Validation	
MolProbity score	2.13
Clash score	16.96
Poor rotamers (%)	0.00
Ramachandran plot	
Favored (%)	94.07
Allowed (%)	5.62
Disallowed (%)	0.31
Model vs Data	
CC (mask)	0.85
CC (box)	0.83
CC (peaks)	0.70
CC (volume)	0.84

Supplementary Table 3: Primers for genetic modification of melbournevirus

ID	Description	Sequence
Av4 F	Add 3'UTR to NEO	GCGGCCGCGGGACTCTGGGGTTCG
Av4 R	Add 3'UTR to NEO	TCAGAAGAACTCGTCAAGAAGGC
Av5 F	Add 3'UTR to NEO	GACGAGTTCTTCTGATTTTTCTTTGTCTCAAAAAAAAGA
Av5 R	Add 3'UTR to NEO	GAGTCCCGCGGCCGCTTTCCATCTCCTTTCTTCAAAAA
Av3 F	Add promoter to NEO	CTTTTGCAAAAAGCTTGATATCTCACTACCTCTATTGCAAG
Av3 R	Add promoter to NEO	TGTGTCAGAAGAATCGATGTCGCACTGCATTTTAGTC
HB1018	KO Melbourn mini histone (5HR)	TGCAAAAAGCTTGATATCGCGGTCTTTGTTGTATCTC
HB1019	KO Melbourn mini histone (5HR)	caaTAGAGGTAGTGAATTACTTTTGGGTGTAAAAAG
HB1020	KO Melbourn mini histone (3HR)	gaaaggAGATGGAAAGGAAAGGGGTAATCTCTGGGAG
HB1021	KO Melbourn mini histone (3HR)	CAGAGTCCCGCGGCCGCGGAGGCCTCTCACTCCTTCG
HB1106	genotype mimihisto melbourn	gtccgtccctcgtgtctt
HB1107	genotype mimihisto melbourn	cgtacgggatcggaactc
HB1114	Add stop to minihistone vector	TGGGAGATAACATATGGTGAGCAAGGGCGAGG
HB1115	Add stop to minihistone vector	ATATGTTATCTCCCAGAGATTACCCCTTTCCC
HB1114	Add stop to minihistone vector	TGGGAGATAACATATGGTGAGCAAGGGCGAGG
HB1115	Add stop to minihistone vector	ATATGTTATCTCCCAGAGATTACCCCTTTCCC

Figures

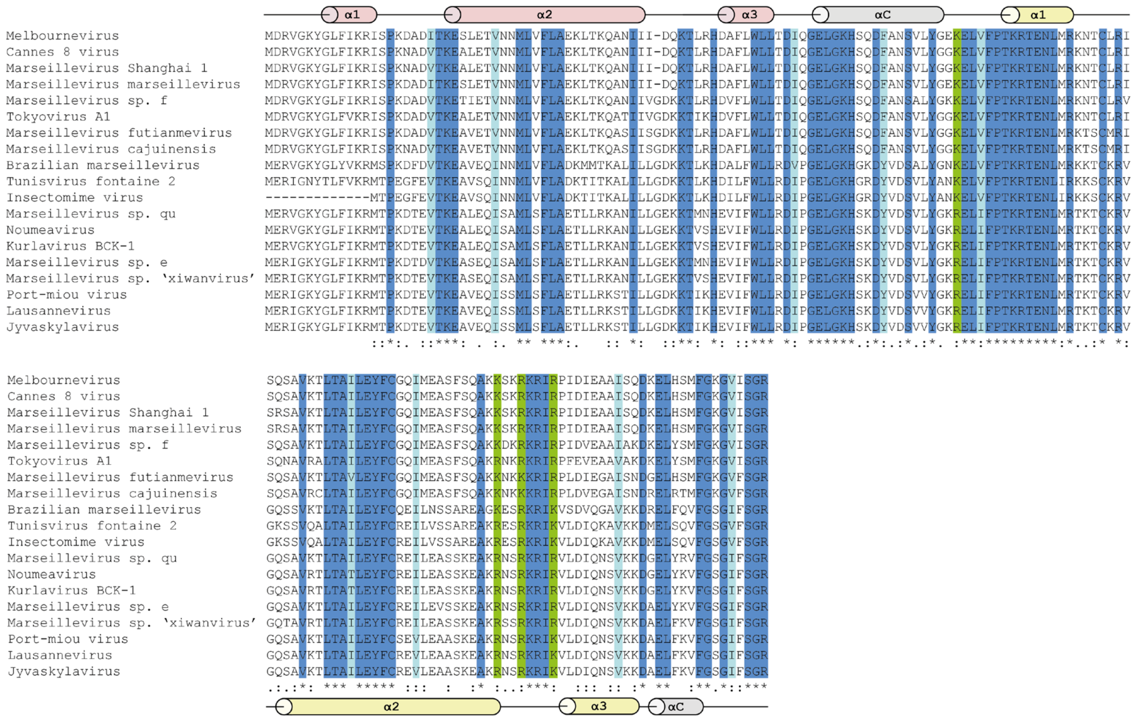

Supplementary Figure 1: The H2B-H2A histone variant is conserved among *Marseilleviridae*. MAFFT[35] alignment of the putative H2B-H2A variant sequences in the family *Marseilleviridae*. Predicted secondary structure elements for melbournevirus protein are shown in light red and light yellow for H2B and H2A regions, respectively. Dark blue highlighted amino acids (aa) are conserved; similar hydrophobic aa are shown in light blue (V/I or F/W/Y), positively charged aa (R/K) are highlighted in green.

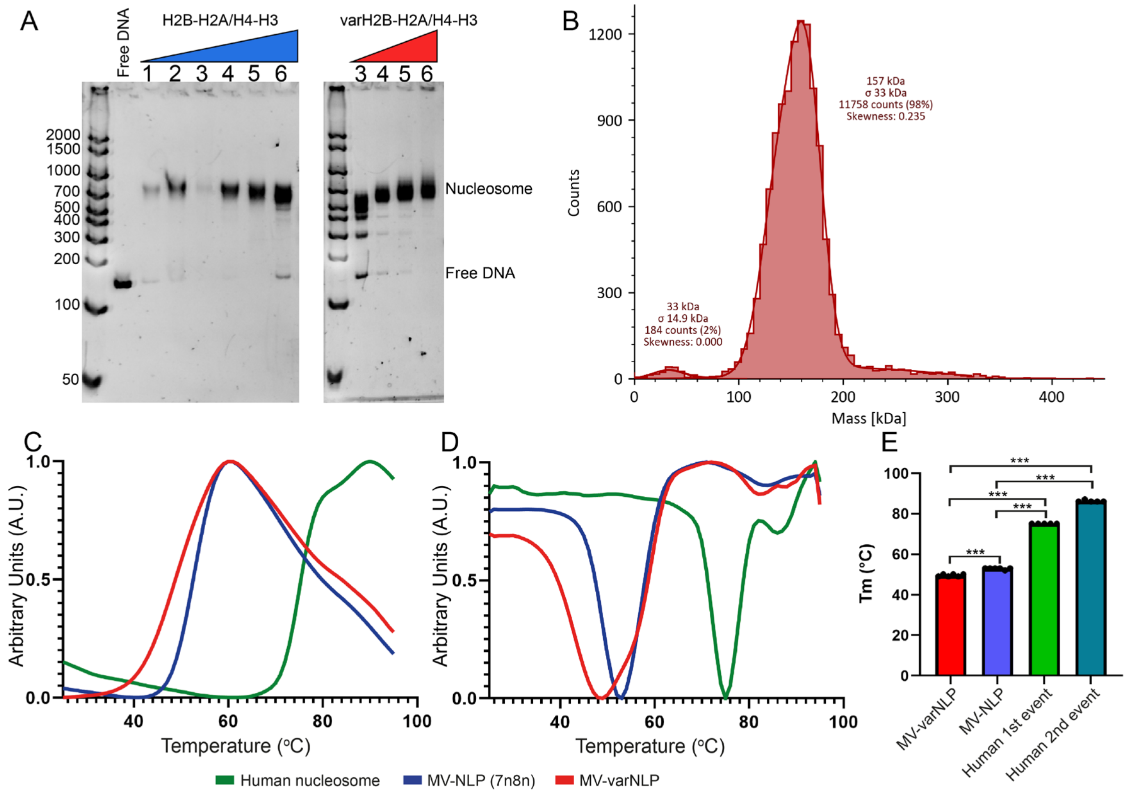

Supplementary Figure 2: MV-varH2B-H2A form nucleosome-like particles. (A) 5% Native PAGE of major and variant nucleosome reconstituted with 147 bp Widom 601 DNA. –DNA: protein ratio: 1= 1:0.9:0.9; 2=1:1:1; 3= 1:1.25:1.25:1.25; 4=1:1.5:1.5; 5=1:1.75:1.75; 6=1:2:2 (major H2B-H2A in blue and H2B-H2A-variant in red). Putative subnucleosomal particles are formed with MV-varH2B-H2A and H4-H3 at low histone-to-DNA ratios. **(B)** Mass Photometry of MV-varNLP (assembled at a 1.6:1 protein:DNA ratio): a homogeneous peak at 157±33 kDa, within error of the calculated molecular weight of MV-varNLP (133.5 kDa) is observed. The minor peak at 33±14.9 kDa is likely from free histones. **(C, D)** Thermal shift assay of MV-varNLP (red), MV-NLP (blue) and human nucleosome (green). **(C)** raw normalized data and **(D)** derivative normalized data. **(E)** Difference (t-test) between melting temperature of MV-NLPs (6 replicates) and human nucleosomes (5 replicates). (***) correspond to a statistically significant difference (p-value <0.0001). Individual points are represented as (•).

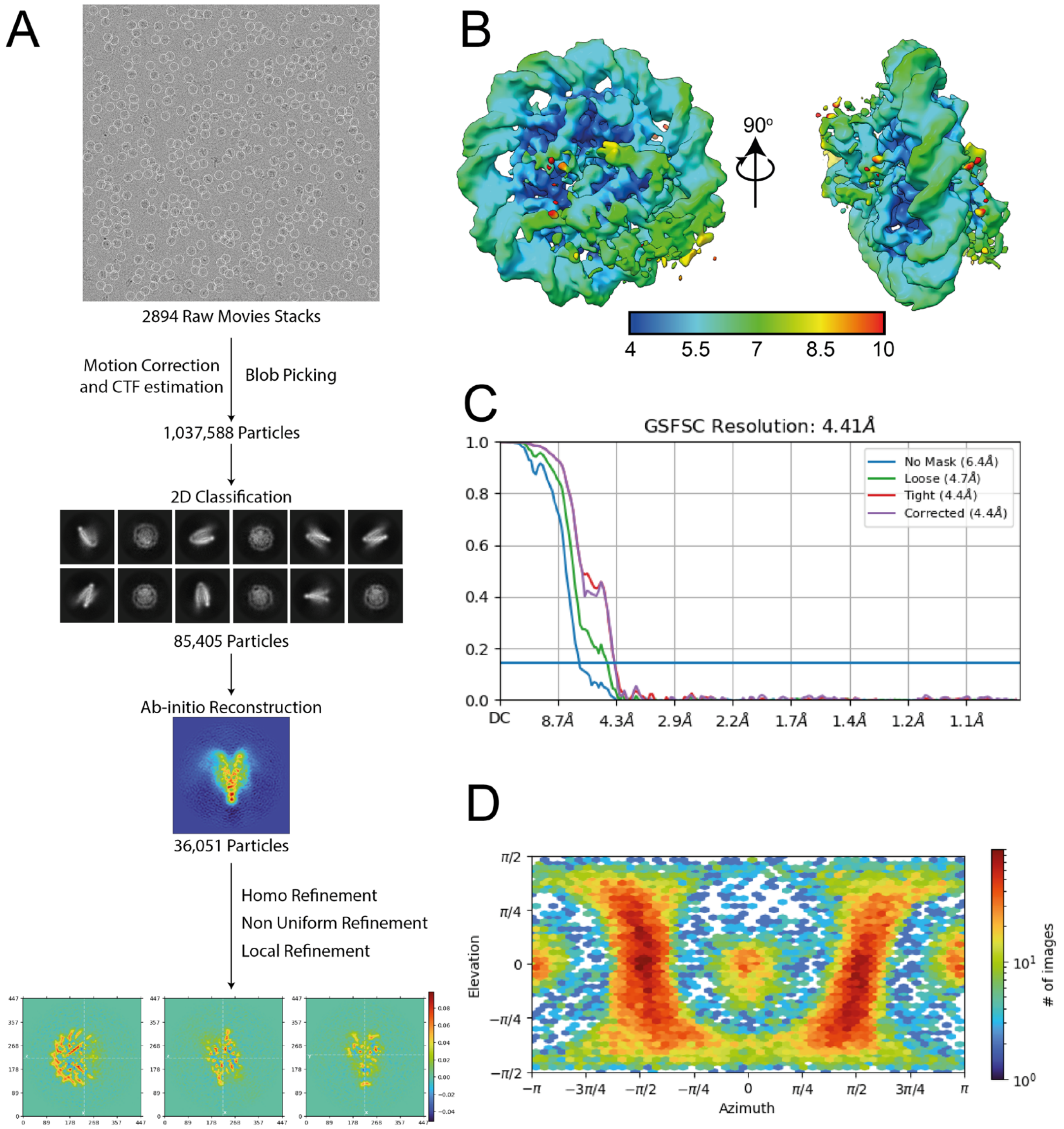

Supplementary Figure 3. Cryo-EM analysis of MV-varNLP.

(A) Single particle analysis CryoSPARC flow chart of MV-varNLP data processing. **(B)** Final map colored by its local resolution. **(C)** FSC curve of MV-varNLP map. Resolution was determined using DSC cutoff of 0.143. **(D)** Azimuth representation of viewing direction distribution.

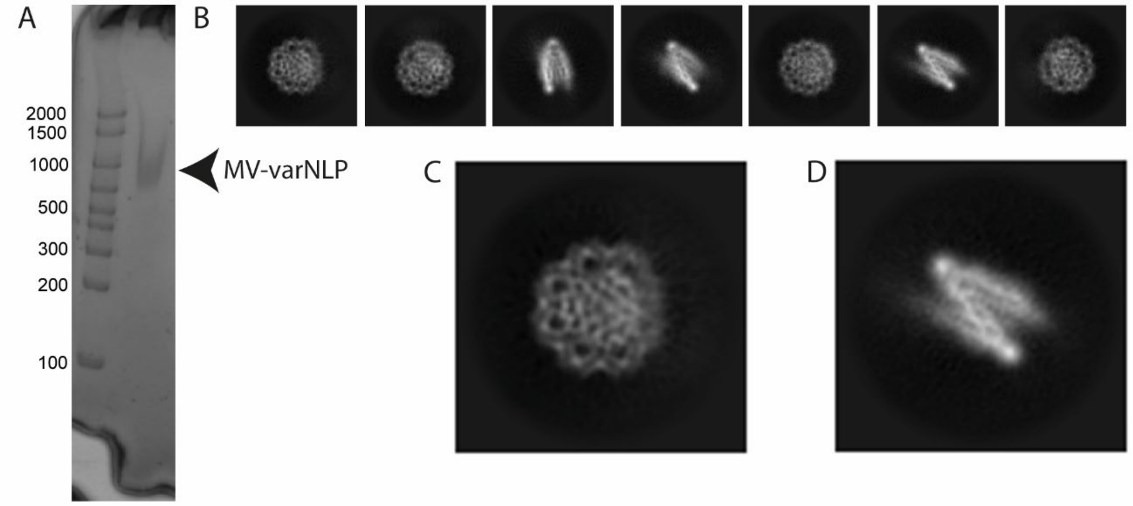

Supplementary Figure 4. MV-varNLP can assemble on a 'random' 150 bp DNA sequence with a GC content of 50 %. (A) 5% TBE native gel demonstrating that the MV-varNLP assembled on random 150 bp DNA sequence forms a nucleosome that migrates identically to MV-varNLP on 601 sequence (compare with Supplementary Figure 2, panel A). B) 2D classes of MV-varNLP assembled on random 150bp DNA sequence, obtained from a small dataset collected on our Titan Krios G3i. C) and D) close-up of two orientations from the 2D classes, illustrating that the overall structure of MV-varNLP is recapitulated with sequences other than the 601 sequence.

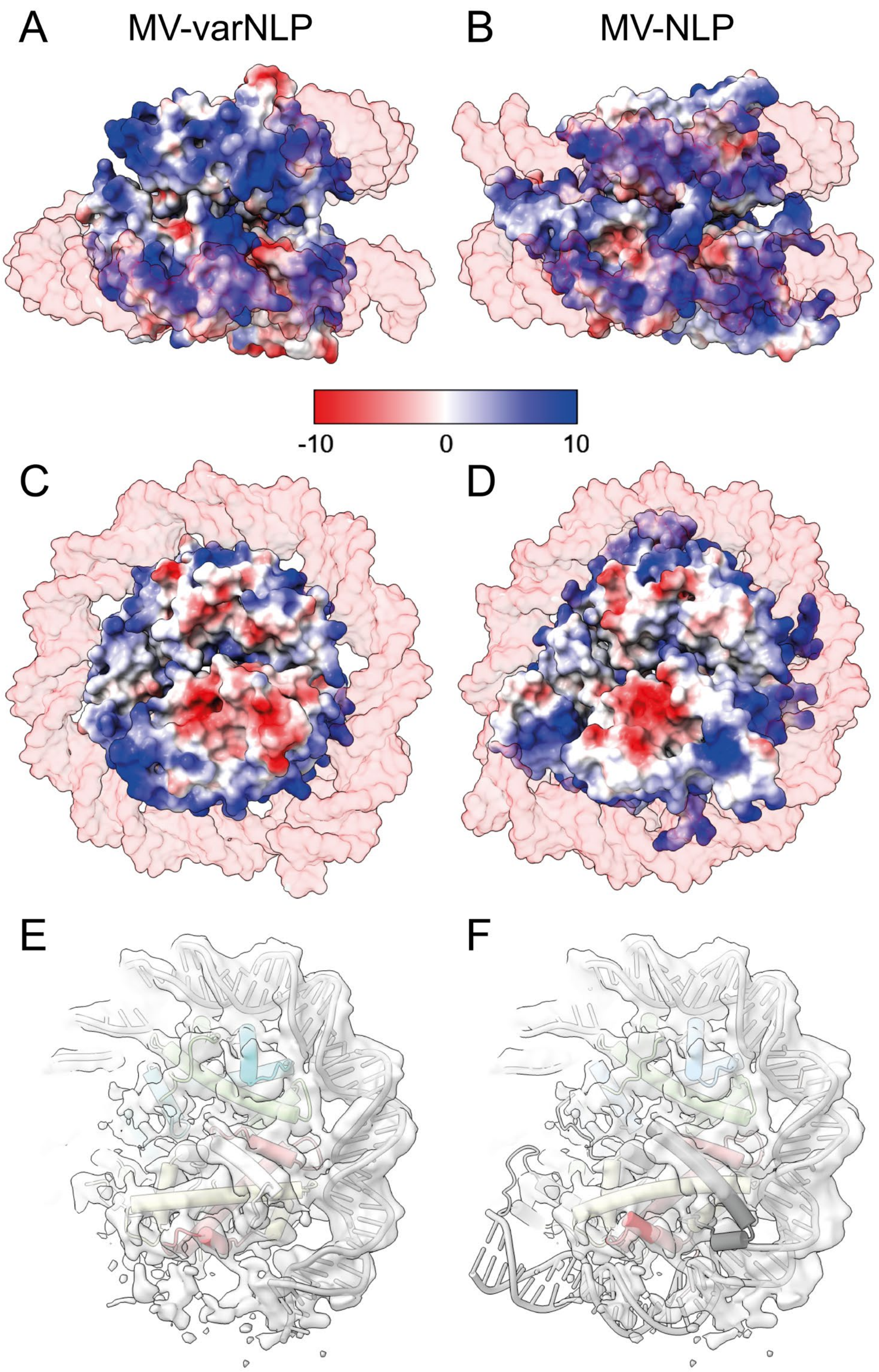

Supplementary Figure 5: Weak density suggests canonical DNA trajectory past SHL ±4.5 in MV-varNLP. Electrostatic surface of **(A, C)** MV-varNLP and **(B, D)** MV-NLP (PDB: 7N8N), Coulombic electrostatic potential values are between -10 kcal/(mol·*e*) and 10 kcal/(mol·*e*) at 298 K. **(E)** MV-varNLP and **(F)** MV-NLP in cartoon model fitted inside the MV-varNLP density. Spurious density for DNA following the canonical superhelical path is visible at low contour levels

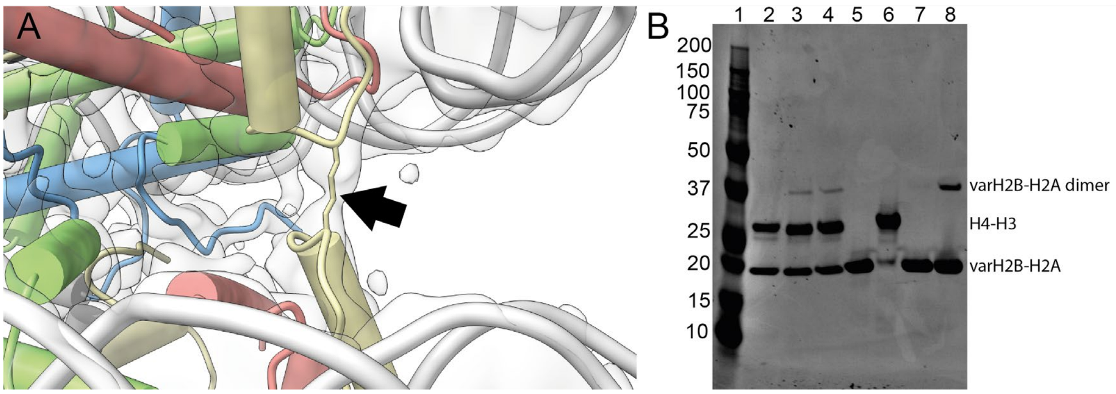

Supplementary Figure 6: MV-varH2B-H2A Cys103 form disulfide bridge stapling together the two varH2B-H2A moieties. (A) Cartoon representation of MV-varNLP (H2A: yellow, H2B: red, H3: blue, H4: green) fitted into its density map. The density for the disulfide bond is indicated by an arrow. **(B)** BisTris 4-12% SDS PAGE. Lane 1: molecular weight marker; 10-200 kDa; 2: MV-varNLP with reducing agent and boiling; 3: MV-varNLP without reducing agent, no boiling; 4: MV-varNLP without reducing agent, with boiling, (5) MV-varH2B-H2A with reducing agent and boiling, (6) MV-H4-H3 with reducing agent and boiling, (7) MV-varH2B-H2A with reducing agent and heat, (8) MV-varH2B-H2A without reducing agent and no heat.

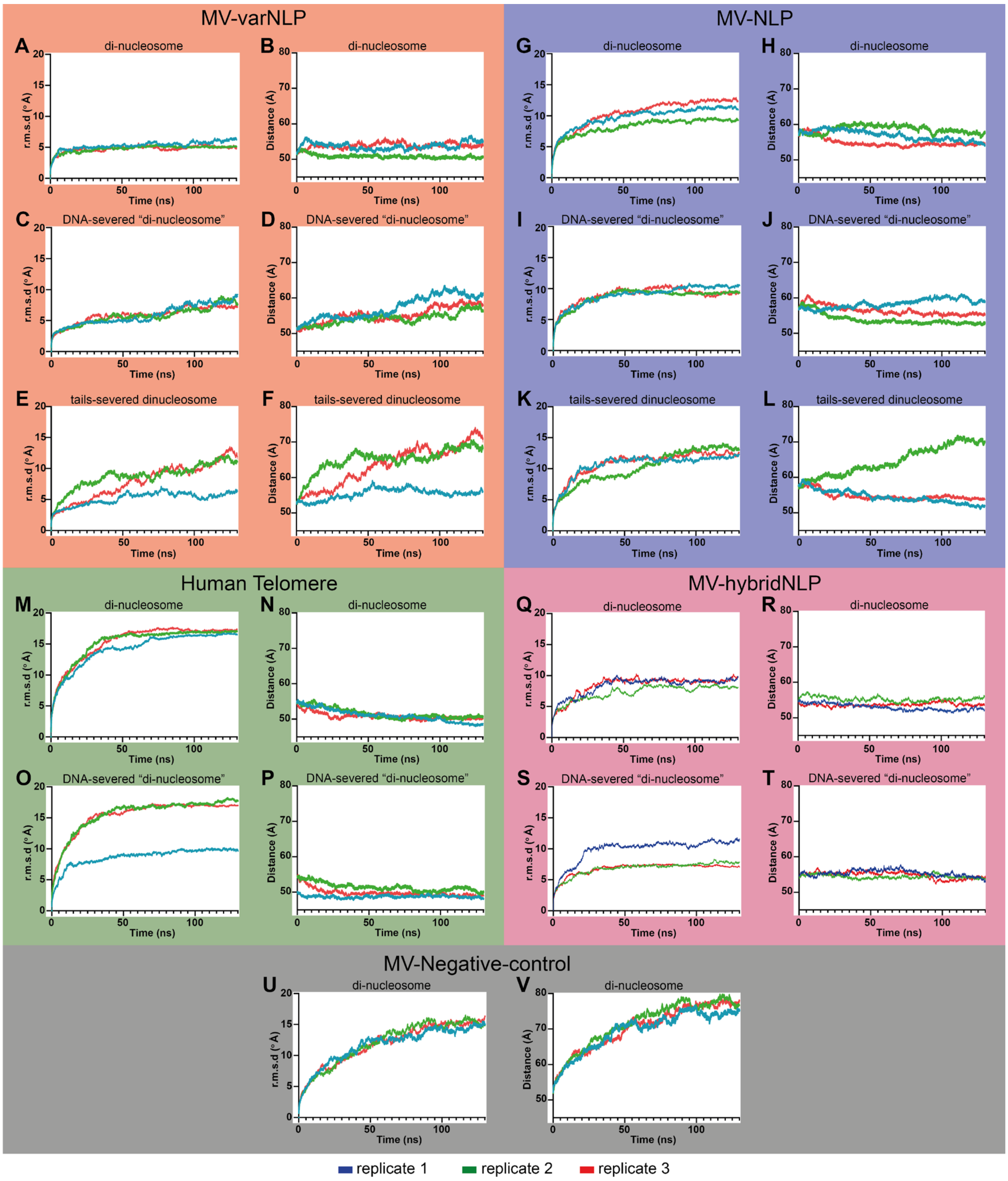

Supplementary Figure 7: Individual molecular dynamics data (all replicates). Left panel for each: root mean square deviation (r.m.s.d.) of the overall structure compared to the start of the simulation; right panel: the distance between the center of gravity of the two nucleosomes in the structure. Individual trajectories of the triplicate molecular dynamic simulations are shown as indicated. For all four types of nucleosomes, we simulated intact di-nucleosomes, as well as di-nucleosomes in which 10 bp of DNA linker was removed (DNA-severed ‘di-nucleosome’). For MV-NLP and MV-varNLP, we also removed the H4 N-terminal tail (MV-H4-H3: deletion of amino acids 1-16). MV-negative-control: MV-varNLP with di-nucleosome contacts mutated to Glu. Individual videos of simulations are uploaded to Figshare (Supplementary videos 1-33).

Videos

Videos are available on figshare

Supplementary video 1: MV-varNLP-rep1

Supplementary video 2: MV-varNLP-rep2

Supplementary video 3: MV-varNLP-rep3

Supplementary video 4: MV-varNLP-DNASevered-rep1

Supplementary video 5: MV-varNLP-DNASevered-rep2

Supplementary video 6: MV-varNLP-DNASevered-rep3

Supplementary video 7: MV-varNLP-TailSevered-rep1

Supplementary video 8: MV-varNLP-TailSevered-rep2

Supplementary video 9: MV-varNLP-TailSevered-rep3

Supplementary video 10: MV-NLP-rep1

Supplementary video 11: MV-NLP-rep2

Supplementary video 12: MV-NLP-rep3

Supplementary video 13: MV-NLP-DNASevered-rep1

Supplementary video 14: MV-NLP-DNASevered-rep2

Supplementary video 15: MV-NLP-DNASevered-rep3

Supplementary video 16: MV-NLP-TailSevered-rep1

Supplementary video 17: MV-NLP-TailSevered-rep2

Supplementary video 18: MV-NLP-TailSevered-rep3

Supplementary video 19: HumanTelomer-rep1

Supplementary video 20: HumanTelomer-rep2

Supplementary video 21: HumanTelomer-rep3

Supplementary video 22: HumanTelomer-DNASevered-rep1

Supplementary video 23: HumanTelomer-DNASevered-rep2

Supplementary video 24: HumanTelomer-DNASevered-rep3

Supplementary video 25: MV-hybrid-rep1

Supplementary video 26: MV-hybrid-rep2

Supplementary video 27: MV-hybrid-rep3

Supplementary video 28: MV-hybrid-DNASevered-rep1

Supplementary video 29: MV-hybrid-DNASevered-rep2

Supplementary video 30: MV-hybrid-DNASevered-rep3

Supplementary video 31: MV-Negative-control-rep1

Supplementary video 32: MV-Negative-control-rep2

Supplementary video 33: MV-Negative-control-rep3

Supplementary information Source Data

Supplementary Figure 2A MV-NLP reconstitution

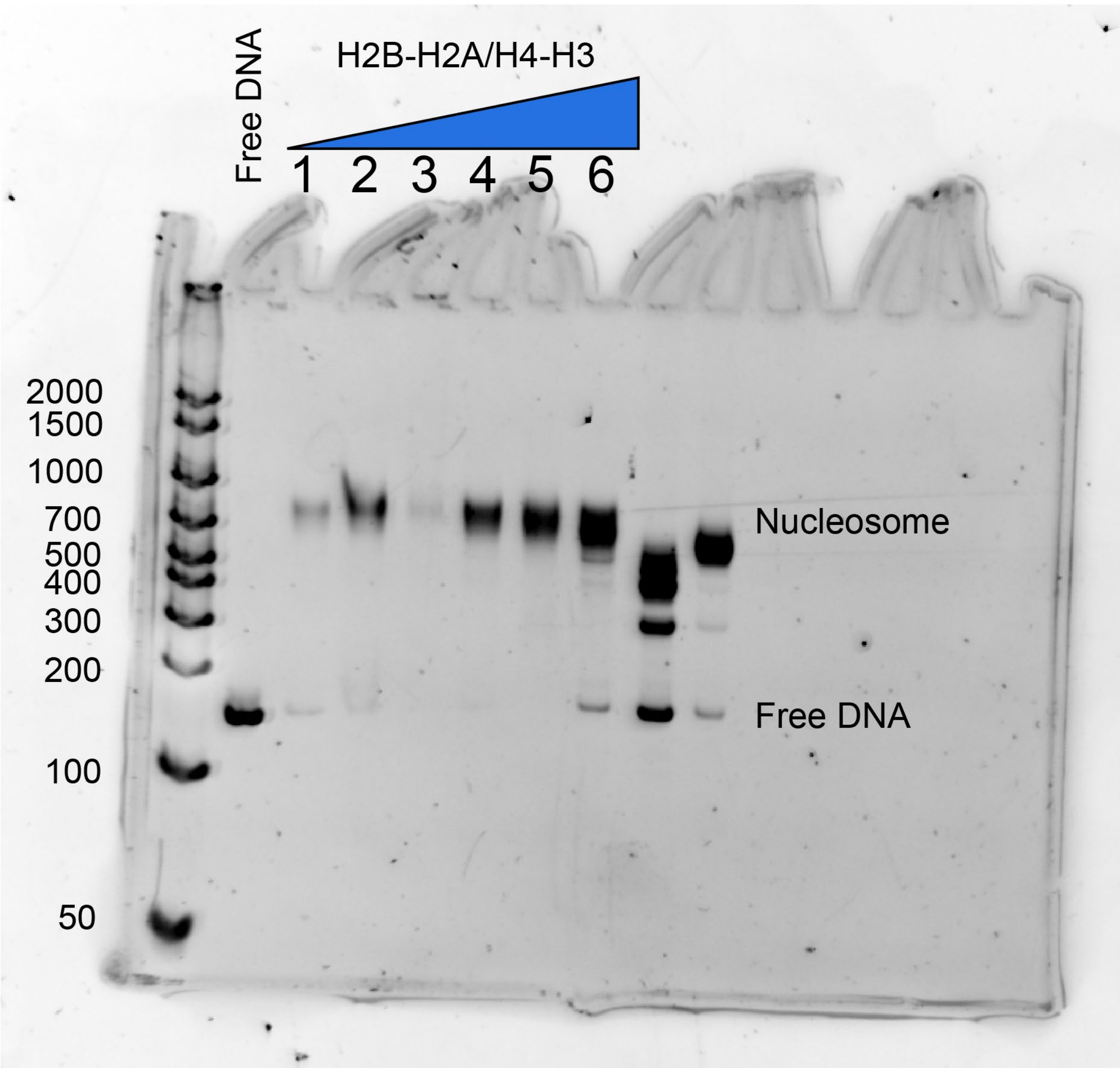

Supplementary Figure 2A MV-varNLP reconstitution

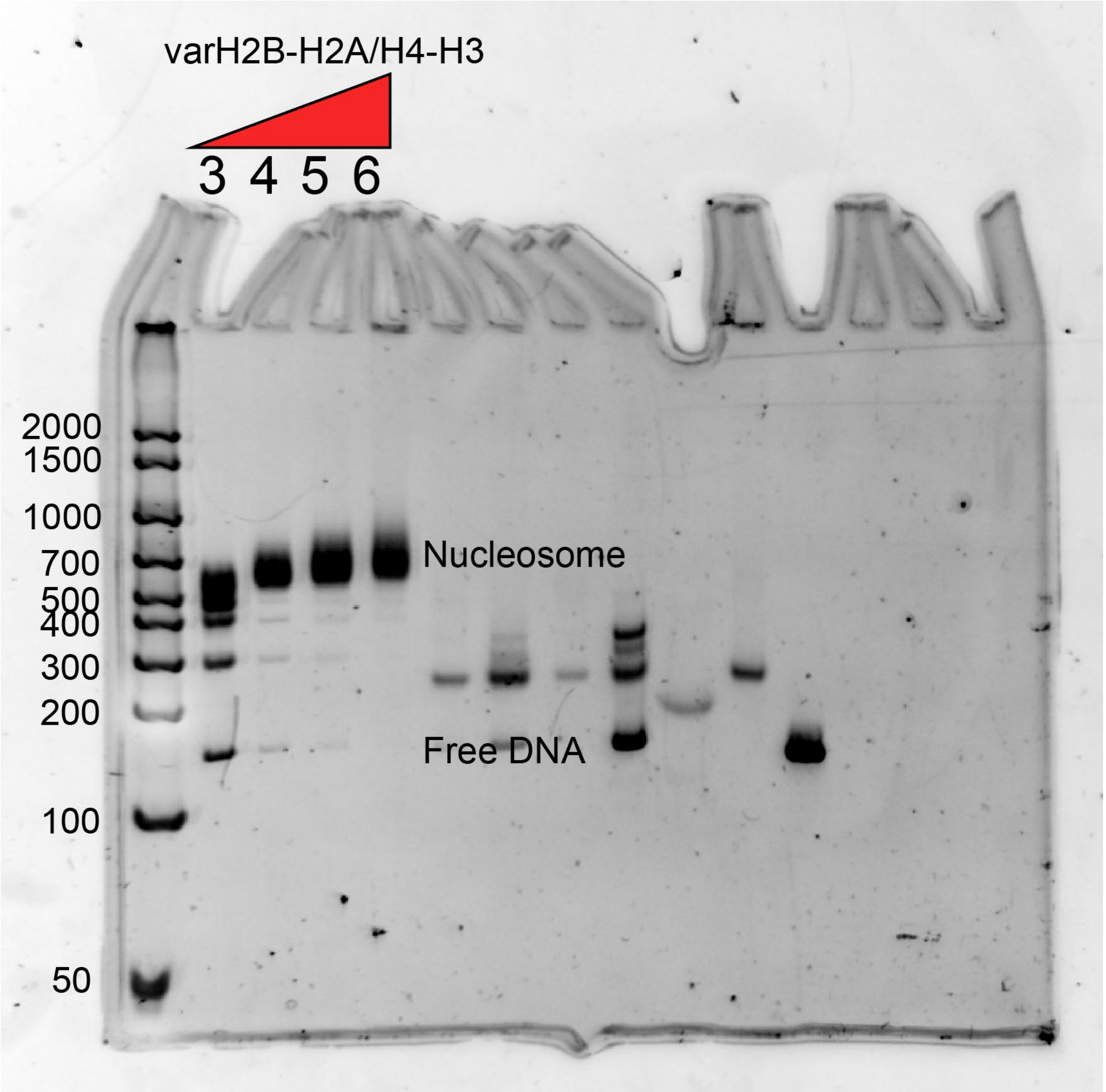

Supplementary Figure 2C Termal Shift Assay raw data

See Source Data file

Supplementary Figure 4A MV-varNLP on random 150 bp DNA sequence

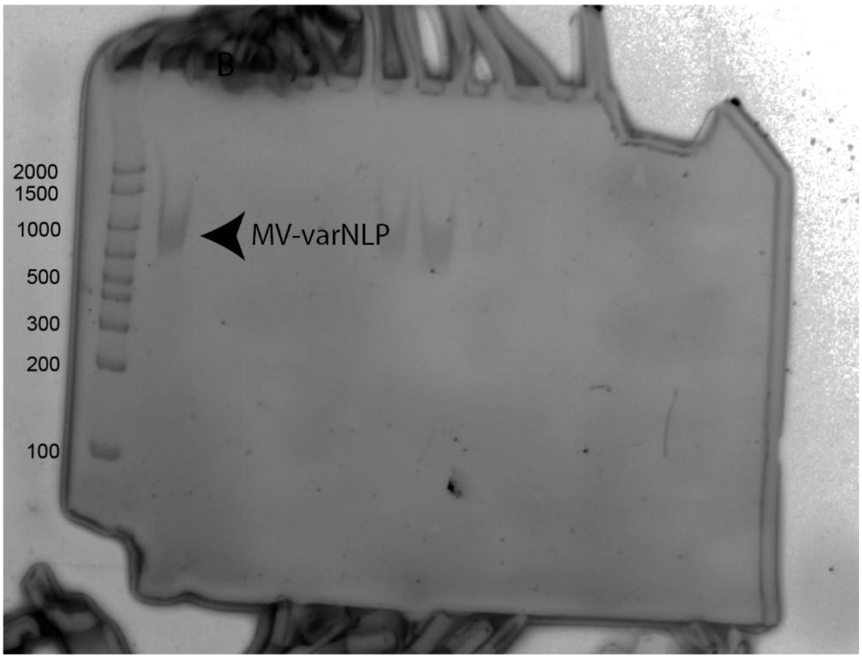

Supplementary Figure 6B MV-varNLP on random 150 bp DNA sequence

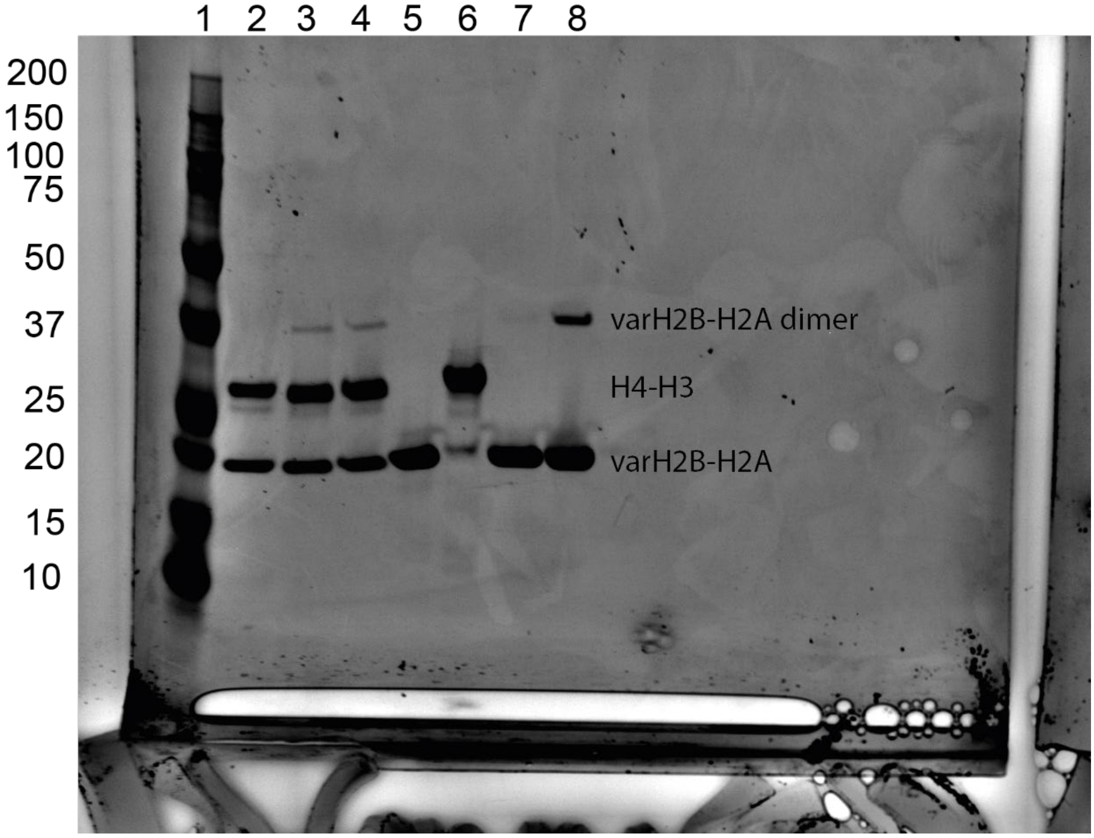

Supplementary Figure 7 Distance and rmsd values

See Source Data file
